# Overview of chronic hepatitis B management

**DOI:** 10.1097/01.NPR.0000000000000246

**Published:** 2024-12-19

**Authors:** Angela Jun, Sherona Bau, John S. Kim, Susanne J. Phillips

**Affiliations:** **Angela Jun** is an associate clinical professor at the Sue and Bill Gross School of Nursing, University of California, Irvine in Irvine, Calif.; **Sherona Bau** is an acute care NP at the Pfleger Liver Institute at the University of California, Los Angeles in Los Angeles, Calif.; **John S. Kim** is a family medicine physician and owner of Cerritos Medical Center in Cerritos, Calif. and a volunteer clinical professor in the School of Nursing, University of California, Los Angeles in Los Angeles, Calif.; **Susanne J. Phillips** is senior associate dean and clinical professor at the Sue and Bill Gross School of Nursing, University of California, Irvine in Irvine, Calif.

**Keywords:** chronic hepatitis B, diagnosis, management, screening

## Abstract

Chronic hepatitis B remains a substantial global health challenge, impacting approximately 254 million people worldwide. A cure for this condition is yet to be discovered. Early identification and effective treatments coupled with vigilant monitoring can help alleviate associated morbidity and mortality due to potential complications such as liver cirrhosis and hepatocellular carcinoma.

**Figure:**
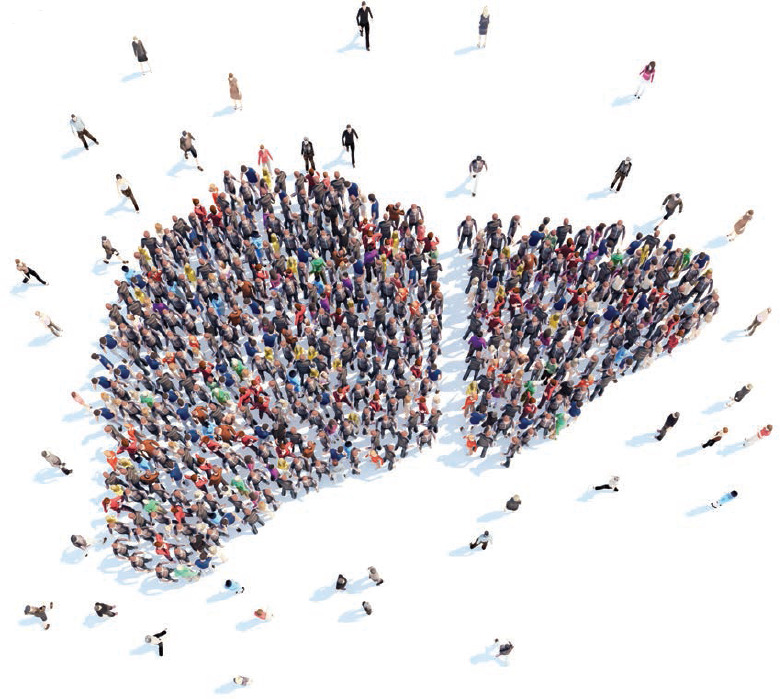
No caption available.

Chronic hepatitis B infection (CHB) remains a global public health challenge. According to the World Health Organization, in 2022, CHB affected 254 million people, leading to an estimated 1.1 million deaths from liver cirrhosis and hepatocellular carcinoma (HCC) worldwide.^1^ According to the 2022 Viral Hepatitis Surveillance Report by the CDC, 16,729 CHB cases were newly reported during the same year.^2^ The age-adjusted death rate per 100,000 population ranged from 0.18 (state of Michigan) to 1.05 (state of Hawaii), with the total number of hepatitis B-associated deaths amounting to 1,797 (an age-adjusted death rate of 0.44 per 100,000) in the US in 2022.^2^ As of now, no cure is known for CHB, but effective treatments with close monitoring can mitigate associated morbidity and mortality.^3^ Early detection of CHB is crucial to reducing the risk of individuals developing severe liver diseases. Therefore, a comprehensive understanding of screening, diagnosis, and pharmacologic management of CHB is essential for primary care NPs to enhance outcomes among patients who are adults, the population under study throughout this article.

## PATHOPHYSIOLOGY AND CLINICAL COURSE

Mature hepatitis B virus (HBV) is a small, enveloped, icosahedral virus with a partially double-stranded, relaxed-circular DNA.^4^,^5^ The main modes of HBV transmission involve percutaneous inoculation or mucosal exposure to infectious body fluids, with oral-fecal transmission being infrequent.^6^ The presence of hepatitis B surface antigen (HBsAg) and other nucleocapsid proteins on hepatocyte cell membranes triggers the T-lymphocyte immune response responsible for the pathogenesis and clinical manifestations of HBV infection.

Most immunocompetent individuals typically recover from acute HBV infection and produce antibodies against HBV. Only a small percentage of individuals progress to CHB, characterized by the sustained presence of HBsAg for more than 6 months, often attributed to impaired cytotoxic T-lymphocyte responses to acute HBV infection.^7^,^8^ Unfortunately, a significant number of CHB cases result from vertical or perinatal transmission from infected mothers to their infants.^8^-^10^ Of individuals infected in this manner, 90% develop CHB; worldwide, vertical or perinatal transmission is responsible for most cases of CHB in adults.

CHB evolves through different clinical phases (immune-tolerant CHB, immune-active CHB, and inactive CHB) in a non-linear pattern that can be reversed. Serologic markers and liver biopsy results vary depending on the phases of CHB (Table 1).^11^,^12^ Although a true cure of CHB (elimination of HBsAg and HBV covalently closed circular DNA [cccDNA]) is not attainable, CHB can rarely be functionally cured, which is characterized by the loss of HBsAg and the acquisition of anti-HBs. However, persistence of HBV cccDNA poses a risk for reactivation of CHB, especially when individuals receive immunosuppressive therapy for other comorbid medical conditions. HBV reactivation is defined by a rise in HBV DNA compared to baseline (or an absolute level of HBV DNA when a baseline is unavailable) and reverse seroconversion (seroreversion) from HBsAg negative to HBsAg positive for HBsAg-negative, anti-HBc–positive patients.^12^ Most individuals with CHB are asymptomatic unless they develop decompensated cirrhosis or extrahepatic symptoms such as polyarteritis nodosa and glomerular disease. The long-term consequences of untreated CHB include liver cirrhosis and HCC, which are associated with high mortality.

**TABLE 1.** Serologic markers and liver biopsy results in different phases of CHB^3^,^12^

Phases of CHB			
	Immune-tolerant CHB	Immune-active CHB	Immune-inactive CHB
**Duration of HBsAg presence**	≥6 months	≥6 months	≥6 months
**HBV DNA**	Very high (>1 million IU/mL)	>20,000 IU/mL in HBeAg-positive CHB and >2,000 IU/mL in HBeAg-negative CHB	<2,000 IU/mL
**HBeAg**	Positive	Negative or positive	Negative
**ALT/AST**	Normal or minimally elevated ALT and/or AST levels	Intermittently or persistently elevated ALT and/or AST levels	Persistently normal ALT and/or AST levels
**Liver biopsy and/or noninvasive testing results**	No fibrosis; minimal inflammation	Moderate or severe necroinflammation; may or may not have fibrosis	Absence of significant necroinflammation; variable levels of fibrosis

Abbreviations: ALT, alanine aminotransferase; AST, aspartate aminotransferase; CHB, chronic hepatitis B infection; HBeAg, hepatitis B e antigen; HBsAg, hepatitis B surface antigen; HBV, hepatitis B virus.

## RISK FACTORS

Certain groups face an increased risk of contracting HBV, including healthcare workers, individuals who use injection drugs, infants born to parents with HBV infection, sexual partners of individuals with HBV infection, individuals undergoing dialysis, and individuals born in countries where HBV infection is prevalent.^3^ Additionally, those with hepatitis C virus (HCV) infection, individuals who have been incarcerated, and those with HIV infection also have an elevated risk. However, it is important to highlight the fact that not all cases of acute HBV infection progress to CHB, as previously mentioned. A few risk factors affect the likelihood of HBV infection progressing to a chronic state.

First, age at the time of infection is one of the most significant risk factors.^13^,^14^ Individuals infected at a younger age face a higher risk of developing CHB. Approximately 9 out of 10 infants who contract HBV go on to experience lifelong, chronic infection.^15^ As a child ages, the risk diminishes, with roughly one in three children infected before age 6 years developing CHB. In contrast, the majority of children ages 6 years and older, as well as adults who acquire HBV infection, typically recover fully and do not experience chronic infection.

Notably, Asian and Pacific Islander (API) people, particularly the non-US-born population, constitute an at-risk population for vertical or perinatal transmission.^2^-^4^,^16^ Despite having the lowest rate of acute HBV infection, the API community experiences a CHB rate 11.2 times higher than that of non-Hispanic White people.^2^-^4^ According to the previously mentioned report from the CDC, in the US, API people exhibit the highest death rate from acute and chronic HBV infection, with a retrospective comparison showing rates of 2.22 (non-Hispanic Asian persons) and 5.19 (non-Hispanic Native Hawaiian or other Pacific Islander persons) per 100,000; the latter is about 19 times the rate among non-Hispanic White persons.^2^ Importantly, the combined rate of death due to HBV infection among API people in 2021 (2.54 per 100,000) was higher than the rate in 2017 (2.45 per 100,000), making API people the only race/ethnicity experiencing an increase in death rates over that time frame.^2^ Literature shows that both a limited understanding and lack of awareness regarding HBV infection and the risk of liver cancer serve as substantial predictors of inadequate monitoring responsible for the negative consequences of CHB across diverse Asian American subgroups.^17^ Moreover, other predictors of adherence to proper monitoring of CHB among this population include psychosocial factors, such as self-efficacy and motivation.

In addition to age at time of infection, specific HBV genotypes may represent a notable factor contributing to an elevated risk of chronicity in HBV infection.^18^,^19^ Among the 10 distinct HBV genotypes (A-J), genotype C in particular seems to be linked to an increased risk of chronicity in HBV infection.

## SCREENING

In 2023, the CDC introduced an updated guideline for HBV infection screening in adults, superseding its 2008 guideline.^3^ The revised guideline advocates for universal triple panel screening, encompassing three serologic tests, to be administered to all adults ages 18 years and older at least once during their lifetime, irrespective of their risk factors (*Box 1*). The triple serologic panel includes HBsAg, antibody to HBsAg (anti-HBs), and total antibody to hepatitis B core antigen (total anti-HBc). HBV screening for all pregnant individuals is recommended during each pregnancy, ideally in the first trimester, regardless of vaccination status or history of testing. However, pregnant individuals with previous appropriately timed triple panel screening and no subsequent risk for exposure to HBV only need HBsAg screening.^3^

Furthermore, the CDC recommends testing for all persons with a history of increased risk for HBV infection, regardless of age, if they might have been susceptible (defined in *Supplemental Box 1*, published online as Supplemental Digital Content available at http://links.lww.com/NPR/A27) during the period of increased risk as well as periodic testing for susceptible individuals with ongoing risk for exposures, while that risk persists.^3^ The updated CDC guideline extends its recommendations for risk-based testing to include individuals incarcerated or formerly incarcerated, individuals with a history of sexually transmitted infections or multiple sex partners, and those with a history of HCV infection, in addition to previously included at-risk groups. This updated guideline addresses the needs of diverse at-risk populations, fostering a more inclusive and effective approach to screening and managing HBV infection. This more inclusive approach may benefit the API population at higher risk of CHB. However, it is important to note that the US Preventive Services Task Force (USPSTF) only recommends screening in adolescents and adults at increased risk for infection.^20^

## DIAGNOSIS

Various serologic markers are available to confirm CHB, and these markers play a crucial role in diagnosis and treatment. However, interpreting the results of these markers can be challenging.^21^ Table 2 and *Supplemental Figure 1* (published online as Supplemental Digital Content available at http://links.lww.com/NPR/A29) provide guidance on determining the different types of HBV infection.^12^,^22^ A persistent presence of HBsAg for more than 6 months is the hallmark of CHB. Upon diagnosing CHB, it is essential to conduct additional tests such as hepatitis B e antigen (HBeAg), which indicates HBV replication and infectivity; HBV DNA; and alanine aminotransferase (ALT) to assess the necessity of treatment. Additionally, screening for HCV and hepatitis D virus (HDV) infections is recommended to rule out superinfection by HCV and HDV.

**TABLE 2.** Interpretation of screening test results for hepatitis B virus infection and recommended actions^3^

Clinical state	HBsAg	Anti-HBs	Total anti-HBc∗	IgM anti-HBc	Action†
Acute infection	Positive	Negative	Positive	Positive	Link to HBV infection care
Chronic infection	Positive	Negative	Positive	Negative§	Link to HBV infection care
Resolved infection	Negative	Positive	Positive	Negative	Counsel about HBV infection reactivation risk
Immune (immunity inferred from receipt of previous vaccination)	Negative	Positive¶	Negative	Negative	Reassure if history of HepB vaccine series completion; if partially vaccinated, complete vaccine series per the ACIP recommendations
Susceptible, never infected	Negative	Negative∗∗	Negative	Negative	Offer HepB vaccine per ACIP recommendations
Isolated core antibody positive‡	Negative	Negative	Positive	Negative	Depends on cause of positive result

Abbreviations: ACIP = Advisory Committee on Immunization Practices; anti-HBs = antibody to hepatitis B surface antigen; HBcAg = hepatitis B core antigen; HBsAg = hepatitis B surface antigen; HBV = hepatitis B virus; HepB = hepatitis B; IgG = immunoglobulin G; IgM anti-HBc = immunoglobulin M antibodies to hepatitis B core antigen; total anti-HBc = total antibody to hepatitis B core antigen.

∗ Total anti-HBc is a measure of both IgM and IgG antibodies to HBcAg.

† Source: Abara WE, Qaseem A, Schillie S, et al. Hepatitis B vaccination, screening, and linkage to care: best practice advice from the American College of Physicians and the Centers for Disease Control and Prevention. *Ann Intern Med* 2017;167:794-804.

§ IgM anti-HBc also might be positive in persons with chronic infection during severe HBV infection flares or reactivation.

¶ Immune if anti-HBs concentration is >10 mIU/mL after vaccine series completion.

∗∗ Anti-HBs concentrations might wane over time among vaccine responders (Source: Schillie S, Vellozzi C, Reingold A, et al. Prevention of hepatitis B virus infection in the United States: recommendations of the Advisory Committee on Immunization Practices. *MMWR Recomm Rep*. 2018;67[No. RR-1]:1-31).

‡ Can be the result of a past infection when anti-HBs levels have waned, occult infection, passive transfer of anti-HBc to an infant born to an HBsAg-positive gestational parent, a false positive, or mutant HBsAg strain that is not detectable by laboratory assay.

Reproduced from: Conners EE, Panagiotakopoulos L, Hofmeister MG, et al. Screening and testing for hepatitis B virus infection: CDC recommendations—United States, 2023. *MMWR Recomm Rep*. 2023;72(No. RR-1):1-25.

Reproduction of this material in the journal does not constitute the journal or publisher's endorsement or recommendation by the US Government, Department of Health and Human Services, or CDC.

## PHARMACOLOGIC TREATMENTS

Initiating pharmacologic treatment for CHB can be confusing due to variations in criteria outlined in current guidelines. The European Association for the Study of the Liver (EASL), American Association for the Study of Liver Diseases (AASLD), and Asian American Treatment Algorithm (AATA) concur on the assessment of ALT and HBV DNA viral load as key criteria to determine the necessity of pharmacologic treatment.^23^,^24^ Table 3 compares CHB treatment initiation criteria from the current EASL, AASLD, and AATA guidelines.^25^

**TABLE 3.** Comparison of treatment recommendations by guideline^24^,^25^

	AASLD	AATA∗	EASL
**Immune-tolerant CHB**	Monitoring is recommended for patients with high HBV DNA (≥10^6^ IU/mL) and normal ALT (males, <35 IU/mL; females, <25 IU/mL) Consider treatment for patients age >40 years with normal ALT, elevated HBV DNA (1,000,000 IU/mL), and liver biopsy showing significant necroinflammation or fibrosis	Insufficient evidence to warrant treatment	Monitoring is recommended for patients with high HBV DNA (≥107 IU/mL) and normal ALT (<40 IU/L) if there are no signs of CHB Treatment may be considered for patients >30 years of age, regardless of the severity of liver histological lesions. Patients with a family history of HCC or cirrhosis and extrahepatic manifestations can be treated.
**Immune-active CHB**	Consider the severity of liver disease to determine whether treatment should be initiated for patients with ALT of 1-2x ULN Treatment is recommended if elevated HBV DNA (≥20,000 IU/mL for HBeAg-positive or ≥2,000 IU/mL for HBeAg-negative CHB) and either ALT ≥2x ULN or evidence of significant histological disease	Reevaluate in 3-6 months if HBV DNA ≤2,000 IU/mL, regardless of ALT level Treatment is recommended if HBV DNA >2,000 IU/mL and elevated ALT Treatment is also recommended if any of the following are present when HBV DNA >2,000 IU/mL and ALT is normal: Albumin <3.5 gm/dL Platelets <130x10^3^ mm^3^ Elevated AFP Basal core promoter mutant HCC in first-degree relative Fibrosis stage ≥2	Treatment is recommended for patients with HBV DNA >20,000 IU/mL and ALT >2x ULN, regardless of the degree of fibrosis Treatment is also recommended for all patients with HBeAg-positive or -negative CHB, defined by HBV DNA >2,000 IU/mL, ALT >ULN, and/or at least moderate liver necroinflammation or fibrosis by biopsy
**Immune-inactive CHB**	Patients with immune-inactive CHB should be monitored	Insufficient evidence to warrant treatment	Patients with immune-inactive CHB should be monitored Consider treatment for patients with HBeAg-negative CHB, family history of HCC or cirrhosis, and extrahepatic manifestations, even if typical treatment indications are not present

Abbreviations: AASLD, American Association for the Study of Liver Diseases; AATA, Asian American Treatment Algorithm; AFP, alpha-fetoprotein; ALT, alanine aminotransferase; CHB, chronic hepatitis B infection; EASL, European Association for the Study of the Liver; HBeAg, hepatitis B e antigen; HBV, hepatitis B virus; HCC, hepatocellular carcinoma; ULN, upper limit of normal.

∗ AATA guideline is intended to apply to Asian American patients.

### Antiviral agents

Peginterferon (peg-IFN) and nucleos(t)ide analogues are useful antiviral drugs for CHB.^12^,^22^,^26^ When individuals are coinfected with HDV, peg-IFN is the medication of choice. However, due to the higher risk of adverse reactions associated with peg-IFN, nucleos(t)ide analogues such as entecavir (ETV) or tenofovir are often preferred. This preference is attributed to the favorable characteristics of these analogues, including tolerability, potent antiviral activity, and low risk of antiviral resistance. This article focuses on nucleos(t)ide analogues, which are potent HBV reverse transcriptase inhibitors.

ETV was approved by the FDA in 2005 for the treatment of CHB. ETV is highly effective with a low resistance rate. The 3-year cumulative probability of ETV resistance was 1.0% and the 5-year cumulative probability was 2.1% in treatment-naive patients in one study.^27^ However, in another study, resistance to ETV was observed in 8.0% of individuals who had exposure to lamivudine but did not have lamivudine resistance and in 28.2% of individuals with lamivudine resistance.^28^ Consequently, AASLD recommends ETV dosing adjustments for patients with lamivudine or telbivudine exposure or decompensated cirrhosis. ETV has a boxed warning for risks of severe acute exacerbations of hepatitis B with discontinuation, lactic acidosis and severe hepatomegaly, and development of resistance to HIV nucleoside reverse transcriptase inhibitors in patients with HIV coinfection that is not being treated. Other potential adverse reactions include, but are not limited to, renal toxicity, skin rash, abdominal pain, and hematuria. Overall, however, adverse reactions from ETV are infrequent.

Tenofovir, which has two formulations (tenofovir disoproxil fumarate [TDF] and tenofovir alafenamide [TAF]), is often used as the initial treatment for patients who are new to treatment and for those who have previously been exposed to or have developed resistance to other nucleos(t)ide analogues (for example, lamivudine).

TDF was approved by the FDA in 2008 for treatment of CHB and is highly effective and well tolerated. In a study of TDF use over 8 years (N = 641), only 41 episodes of virological breakthrough occurred throughout the study.^29^ Of those, 70% were associated with nonadherence to the treatment regimen. TDF has a boxed warning for risk of posttreatment acute exacerbation of hepatitis B. Potential adverse reactions include insomnia, headache, dizziness, skin rash, hypercholesterolemia, decreased bone density, and increased serum creatinine, among others.

TAF, approved by the FDA in 2016 for treatment of CHB, is equally as effective as TDF and has the same boxed warning for posttreatment acute exacerbation of hepatitis B. In a phase III clinical trial in 2018, 64% of participants achieved HBV DNA levels of less than 29 IU/mL, and 72% of participants saw ALT levels return to normal after 48 weeks of treatment.^30^ HBsAg loss, however, was less than 1%. Fatigue (6%), nausea (6%), and headache (12%) were among the most common adverse reactions. TAF seems to have less renal and bone toxicity than TDF. It is therefore a better choice for initial treatment in older patients and those with risk factors for renal impairment or osteoporosis.

### Treatment goals and monitoring

Currently, a virologic cure (true cure) characterized by elimination of HBsAg and of HBV cccDNA for CHB does not exist.^11^ However, functional cure, defined as undetectable HBsAg and HBV DNA in serum with seroconversion to anti-HBs after 6 months off therapy, can be an attainable goal of pharmacologic therapy.^31^ Functional cure is known to be associated with normalization of serum ALT and improved liver histology.

For treatment with nucleos(t)ide analogues, the recommended duration of therapy varies among guidelines.^25^ AASLD recommends indefinite antiviral therapy for adults with HBeAg-negative immune-active CHB, unless reasons for treatment discontinuation are compelling.^12^ The AASLD guideline provides detailed information regarding this recommendation as well as CHB therapy recommendations for patients in other phases and other specific situations.^12^ It is noteworthy that even after attaining a functional cure individuals remain at risk for HBV infection reactivation upon termination of treatment due to the persistent presence of HBV cccDNA in the nuclei of liver cells.^11^,^31^

Monitoring while on pharmacologic treatment involves assessing various parameters to evaluate potential adverse reactions.^12^,^32^ All nucleos(t)ide analogues require renal dosing adjustment; therefore, prior to initiating this treatment, checking serum creatinine and estimated creatinine clearance is recommended.^12^,^32^ If a patient is at risk for renal impairment or on TDF, creatinine clearance and serum phosphorus should be checked every 3 months for the first year of treatment and every 6 months thereafter once renal function is stable.^32^

In addition, if there is any clinical concern for heart failure or sepsis while on the treatment, lactic acid levels should be monitored.^12^ If a patient on TDF has a history of fracture or is at risk for osteopenia, a bone density study should be considered at baseline and during treatment.^12^,^32^ Quantitative HBV DNA testing and ALT level checks are essential prior to and during treatment; both should be checked every 3 months initially until normalization of ALT and achievement of an undetectable level of HBV DNA. Thereafter, levels should be checked every 3 to 6 months. Given the high prevalence of HBV and HIV coinfection, along with the increased risk of HCC and all-cause mortality in these individuals, it is recommended that clinicians test for HIV before initiating treatment with nucleos(t)ide analogues for proper treatment.^12^,^33^ Special treatment considerations for patients with HBV and HIV coinfection can be found in the guidelines and drug package inserts.

In addition, periodic monitoring for the development of HCC is recommended in certain patients, irrespective of whether they receive antiviral therapy.^14^ The guidelines provide slightly different recommendations in this regard. Ultrasound screening every 6 months, with or without screening for alpha-fetoprotein, seems reasonable and is recommended for the following populations: 1) all HBsAg-positive patients with cirrhosis and 2) HBsAg-positive adults at high risk for HCC, such as Asian men older than 40 years of age, Asian women older than 50 years of age, Black men and women, persons with a first-degree family member with a history of HCC, and persons coinfected with HDV or HIV.^12^,^23^,^24^

## NP PRACTICE IMPLICATIONS AND CONCLUSION

This article reviews the 2023 hepatitis B screening guideline from the CDC as well as CHB diagnosis and treatment, including the differences among various organizations' clinical practice guidelines regarding pharmacologic therapy considerations. Primary care NPs can play a pivotal role in identifying and addressing CHB with vigilant screening as recommended by the new hepatitis B screening guideline from the CDC and by becoming familiar with the diagnostic algorithm and possible treatment options. Active engagement in educating individuals about their condition, emphasizing the importance of regular follow-ups, and collaborating with other healthcare providers when necessary are crucial. The goal of care is not only to manage the immediate impact of CHB but also to contribute to long-term health outcomes and mitigate the risk of disease progression and complications such as liver cirrhosis and HCC. Regularly referring to updated guidelines and staying informed about advancements in CHB management ensure that NPs provide care aligned with evolving standards in this field.
